# E/Ea ratio could not predict fluid response in ICU mechanically ventilated patients

**DOI:** 10.1186/cc9477

**Published:** 2011-03-11

**Authors:** J Cousty, A Mari, P Marty, B Riu, P Sanchez, O Mathe, J Ruiz, S Silva, F Vallée, M Génestal, O Fourcade

**Affiliations:** 1 Reanimation polyvalente CHU Purpan, Toulouse, France; 2 CHU Purpan, Toulouse, France

## Introduction

Transthoracic echocardiography (TTE) is now widely used in the ICU to assess hemodynamic status. Combined mitral index measured by TTE, as the mitral Doppler inflow E wave velocity to annular tissue Doppler Ea wave velocity ratio (E/Ea), is a reliable diastolic indicator in cardiologic patients. In ICU, E/Ea has only been investigated as a pulmonary arterial occlusion pressure surrogate which poorly reflects fluid responsiveness (FR). Therefore, the aim of this study was to evaluate the reliability of E/Ea to FR in the setting of ICU ventilated patients.

## Methods

We carried out a TTE prospective observational study in mechanically ventilated patients receiving fluid challenge for circulatory failure. Complete TTE examination involving stroke volume (SV) estimation, mitral and tissue Doppler measurements (E, A, Ea, Aa velocities) were performed at end-expiratory time, before and after a 500 ml saline solution over 15 minutes of fluid challenge. A positive hemodynamic response was defined as a 15% minimal increment of SV. General characteristics, mitral parameters and combined index (E/A and E/Ea) were compared between responders (R) and nonresponders (NR) (using Student *t *test or chi-square test, ROC analysis and LHR method).

## Results

Ninety-four case-mix patients were enrolled: 43 R and 51 NR, with similar baseline characteristics. LV ejection fraction was: altered (< 50%) *n *= 24, or preserved (> 50%) *n *= 69, with no difference (R vs. NR). E/Ea values before fluid loading were not statistically different between R and non-NR for which we observed a huge overlap (7.4 ± 2.4 vs. 8.4 ± 3.1 R vs. NR; *P *= 0.09). The results were similar when considering the population with baseline under the median value; that is, E/Ea <8: 28 R versus 24 NR, E/Ea = 6.0 ± 1.5 versus 5.6 ± 1.5 R versus NR, *P *= 0.28. The E/A index was significantly lower in R (1.1 ± 0.4 vs. 1.3 ± 0.4; *P *< 0.01) but poorly predicted FR: ROC curve AUC = 0.64 (0.54 to 0.74), best cut-off: 0.8 (LHR+ 3.1; LHR- 0.7). Extreme values were predictive in our population: R was likely with E/A <0.6 (Sp 100%, LHR+ >5) and unlikely with E/A >1.8 (Se 100%, LHR- <0.2).

## Conclusions

The E/Ea ratio is not statistically different between responders and nonresponders in the ICU and no low discriminant threshold value of E/Ea could identify patients likely to respond to fluid expansion. While E/A is statistically significant, only extreme values could be clinically relevant (< 0.6 or >1.8).

